# Apparent Diffusion Coefficient (ADC) predicts therapy response in pancreatic ductal adenocarcinoma

**DOI:** 10.1038/s41598-017-16826-z

**Published:** 2017-12-06

**Authors:** M. Trajkovic-Arsic, I. Heid, K. Steiger, A. Gupta, A. Fingerle, C. Wörner, N. Teichmann, S. Sengkwawoh-Lueong, P. Wenzel, A. J. Beer, I. Esposito, R. Braren, J. T. Siveke

**Affiliations:** 1 Division of Solid Tumor Translational Oncology, West German Cancer Center, University Hospital Essen, Essen, Germany; 2 German Cancer Consortium (DKTK, partner site Essen) and German Cancer Research Center (DKFZ), Heidelberg, Germany; 3 Institute of Radiology, Klinikum Rechts der Isar, Technical University of Munich, Munich, Germany; 4 Institute of Pathology, TUM School of Medicine, Technical University of Munich, Munich, Germany; 5 2. Medizinische Klinik, Klinikum Rechts der Isar, Technical University of Munich, Munich, Germany; 6 Department of Nuclear Medicine, Klinikum Rechts der Isar, Technical University of Munich, Munich, Germany; 7 Present Address: Department of Nuclear Medicine, University Hospital of Ulm, Ulm, Germany; 8 Institute of Pathology, University Clinic Duesseldorf, Heinrich-Heine University, Duesseldorf, Germany

## Abstract

Recent advances in molecular subtyping of Pancreatic Ductal Adenocarcinoma (PDAC) support individualization of therapeutic strategies in this most aggressive disease. With the emergence of various novel therapeutic strategies and neoadjuvant approaches in this quickly deteriorating disease, robust approaches for fast evaluation of therapy response are urgently needed. To this aim, we designed a preclinical imaging-guided therapy trial where genetically engineered mice harboring endogenous aggressive PDAC were treated with the MEK targeting drug refametinib, which induces rapid and profound tumor regression in this model system. Multi-parametric non-invasive imaging was used for therapy response monitoring. A significant increase in the Diffusion-Weighted Magnetic Resonance Imaging derived Apparent Diffusion Coefficient (ADC) was noted already 24 hours after treatment onset. Histopathological analyses showed increased apoptosis and matrix remodeling at this time point. Our findings suggest the ADC parameter as an early predictor of therapy response in PDAC.

## Introduction

Treatment of Pancreatic Ductal Adenocarcinoma (PDAC) is suffering from largely ineffective therapeutic regimens^1^. However, intensified chemotherapeutic protocols result in increased response rates and may even lead to complete pathological remission in individual cases demonstrating heterogeneity of therapy responses^2,3^. Furthermore, advancements in our understanding of PDAC and subtyping of cancers potentially amendable to differential treatment strategies emphasizes the importance of individualized approaches and early evaluation of therapy effectiveness^4,5^.

Aside from anatomical information, at current no imaging modality has a prognostic or predictive role in clinical decision making^6–8^. For therapy evaluation, routine imaging protocols mostly rely on Computed Tomography (CT) or Magnetic Resonance Imaging (MRI)^9,10^. Despite the prominent role of CT imaging in diagnosis of PDAC, the complex microarchitecture and strong desmoplasia in PDAC are considerable challenges for reliable delineation of tumor margins and thus tumor volume and therapy response assessment. We recently showed that tumor cellularity is a prognostic feature^11^ and may be of predictive value given the emergence of stroma-acting treatment approaches. Therapy-induced morphological or cellular changes that are potentially relevant in neoadjuvant settings cannot easily be detected by structural imaging such as CT or T1-/T2-weighted-Magnetic Resonance Imaging (MRI). These limitations clearly necessitate better imaging based markers for therapy response.

[^18^F]-Fluordeoxyglucose (FDG) Positron Emission Tomography (PET) is also evaluated for diagnosis and staging of PDAC with however controversial results^12,13^. Cancers, including PDAC, are often highly glucose dependent and reports suggest a positive correlation between [^18^F]-FDG glucose Standard Uptake Values (SUV) and tumor size thus qualifying FDG-PET as treatment response marker^13,14^.

Diffusion Weighted MR Imaging (DW-MRI) is increasingly used in cancer diagnosis and follow-up, appealing as a non-invasive procedure that does not require use of contrast agent of ionizing radiation. DW-MRI measures diffusion of water molecules in the tissue and is influenced by tissue composition including cellularity, stroma content and vascularization^15^. Tissue parts of different cellular density and stromal content are thus characterized by different Apparent Diffusion Coefficients (ADC) reflecting the freedom of water molecules movements.

DW-MRI has been evaluated for PDAC staging^16^, differentiation of pancreatitis from PDAC^17^, tumor identification in high risk individuals^18^ and as therapy response parameter^19,20^.

We thus aimed to further investigate the role of DW-MRI in early therapy response using a spontaneous complex mouse model of PDAC. Conditional oncogenic Kras mouse models developing endogenous PDAC recapitulate the complex tumor composition, aggressive tumor behavior and desmoplastic reaction and have consequently been used in various preclinical therapy trial settings including MR imaging guided trials also from our group^21–23^. We here report on multimodal and multi-parametric non-invasive imaging in genetically engineered mice (GEM) with endogenous aggressive PDAC treated with standard of care chemotherapy gemcitabine and the Mitogen-activated protein kinase kinase (MEK) targeting drug refametinib. Drug response was followed with [^18^F]-FDG-PET, T2-weighted and DW-MRI.

## Results

### MAP kinase inhibition leads to decrease in tumor volume

**Figure 1:**
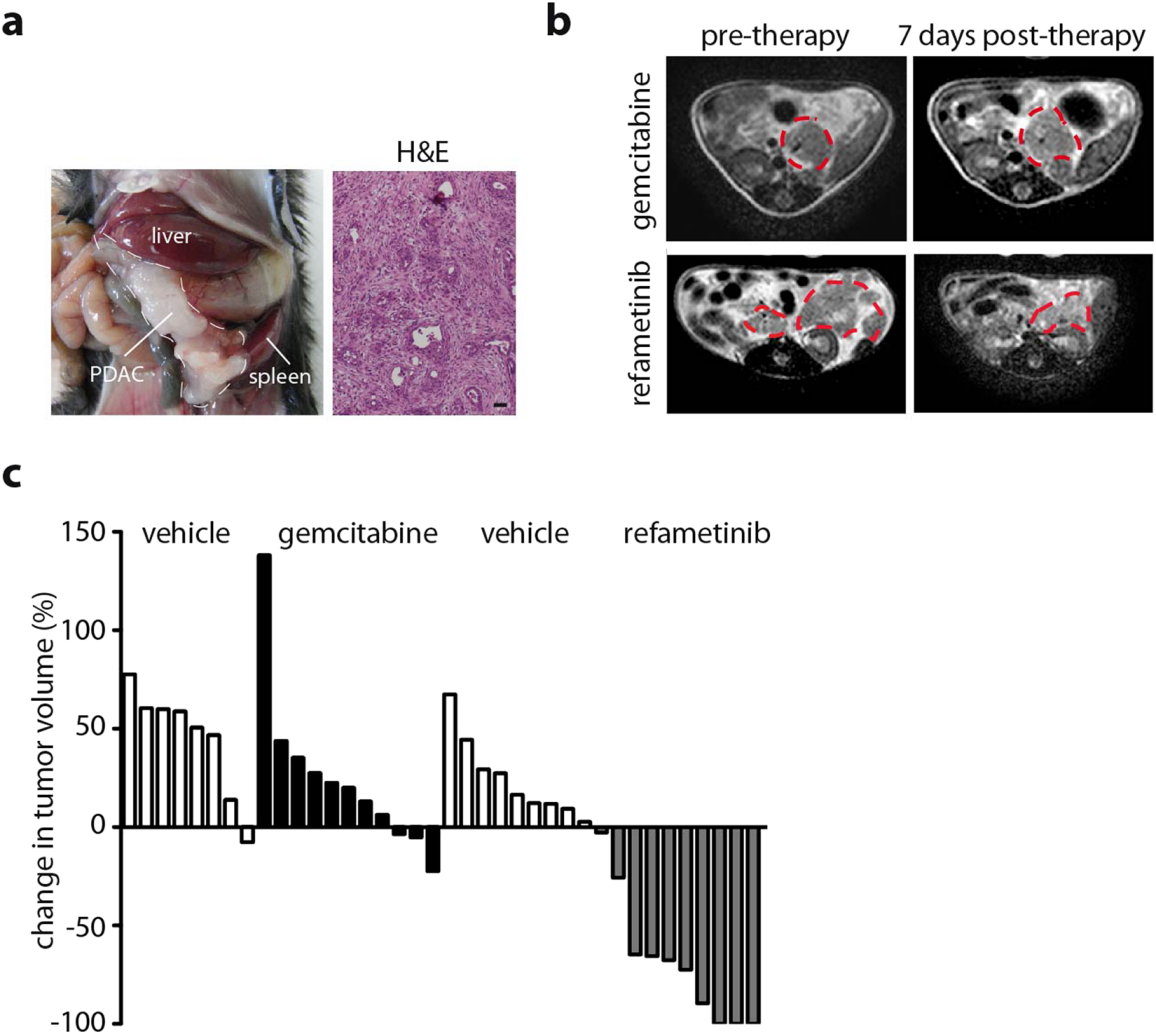
MEK inhibition but not gemcitabine elicits a tumor response in CKP mice. (**a**) Photograph of a CKP animal showing a locally advanced PDAC and H&E staining demonstrating typical histological appearance of PDAC with epithelial-glandular differentiation and strong desmoplastic reaction. (**b**) Representative T2w images before and after therapy initiation with gemcitabine or refametinib. Tumor is delineated with a dotted line. A decrease in tumor size is visible in refametinib but not gemcitabine treated animals. (**c**) Waterfall plot of tumor volume change showing no response in Gemcitabine and significant response in refametinib treatment group (Gemcitabine: 7 days upon treatment onset, 2 doses in total; refametinib: 5–9 days upon treatment onset, 5–7 doses in total). Tumor volumes calculated based on T2w-MR images and expressed relative to the pre-treatment tumor volume. Each column represents one mouse.

*Ptf1a* ^*wt/cre*^; *Kras* ^*wt/LSL-KrasG12D*^; *p53* ^*fl/fl*^ mice (referred to as *CKP* hereafter) were used as a model of locally advanced PDAC that exhibits a typical human-like morphology with abundant desmoplasia, moderate to poor epithelial differentiation and a highly aggressive clinical course (Fig. 1a)^24,25^. As previously shown for this model^22^, gemcitabine treatment led to no obvious tumor volume responses inducing at best a low-frequent effect (Fig. 1b,c). In contrast, treatment with the MEK inhibitor refametinib resulted in dramatic tumor shrinkage in virtually all animals only 5 to 9 days post treatment onset (>50% reduction in tumor volume in 80% of mice) as calculated from T2w-MRI data (Fig. 1b,c) supporting a suitable model system to study early response monitoring of treatment effects.

### DW-MRI but not FDG-PET detects early therapy induced changes

**Figure 2:**
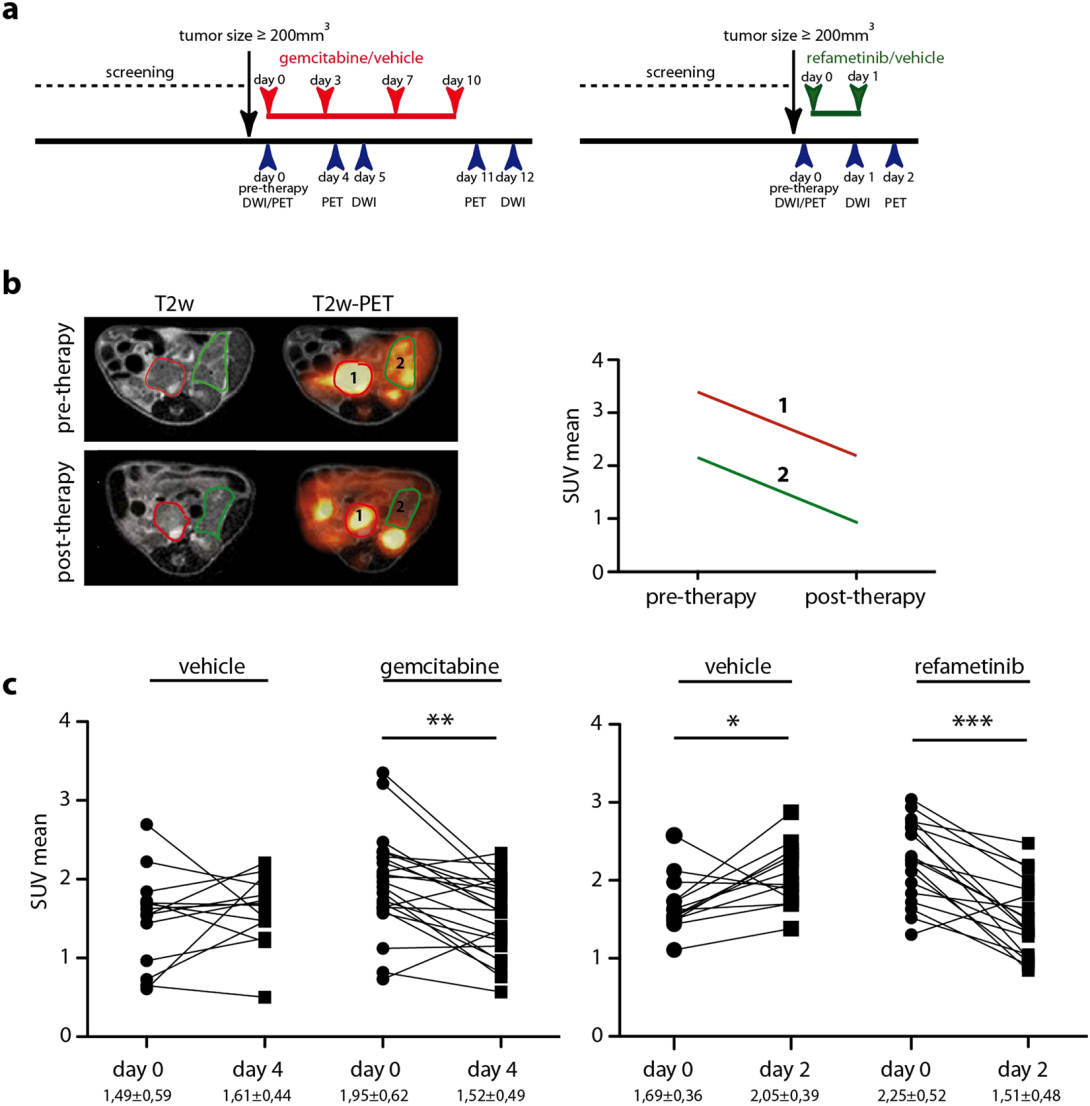
[^18^F]-FDG PET analysis in gemcitabine/refametinib treated GEM. (**a**) Schematic representation of treatment and imaging plan for gemcitabine/vehicle and refametinib/vehicle treated CKP mice. Animals were weekly scanned and once the tumor reached the size of 200 mm^3^ or bigger, animals were enrolled into the study. (**b**) Exemplary presentation of PET analysis: T2w and corresponding PET (fused) images before and after the therapy demonstrating presence of two tumors with different [^18^F]-FDG uptake in the same animal (one delineated with red and another delineated with green line). Same tumor was followed pre- and post-therapy and SUV values were calculated (right graph). (**c**) Decrease in SUV mean values observed upon short-term gemcitabine and refametinib treatment. One line presents one tumor followed before and after therapy. Vehicle p = 0,46; gemcitabine p = 0,0013; vehicle p = 0,03; refametinib p = 0,0007.

To evaluate [^18^F]-FDG-PET and DW-MRI for early response imaging, we set up a short-term treatment protocol with gemcitabine or refametinib in CKP mice (Fig. 2a). Animals received all imaging modalities prior to therapy and early post therapy onset. At baseline, we observed high metabolic heterogeneity with varying intra- and intertumoral FDG-uptake (Fig. 2b). However, a decrease in SUV values was observed in both therapy groups, gemcitabine and refametinib treated animals, independent of the anti-tumor effect (Fig. 2c, Supplementary Figure 1).

**Figure 3:**
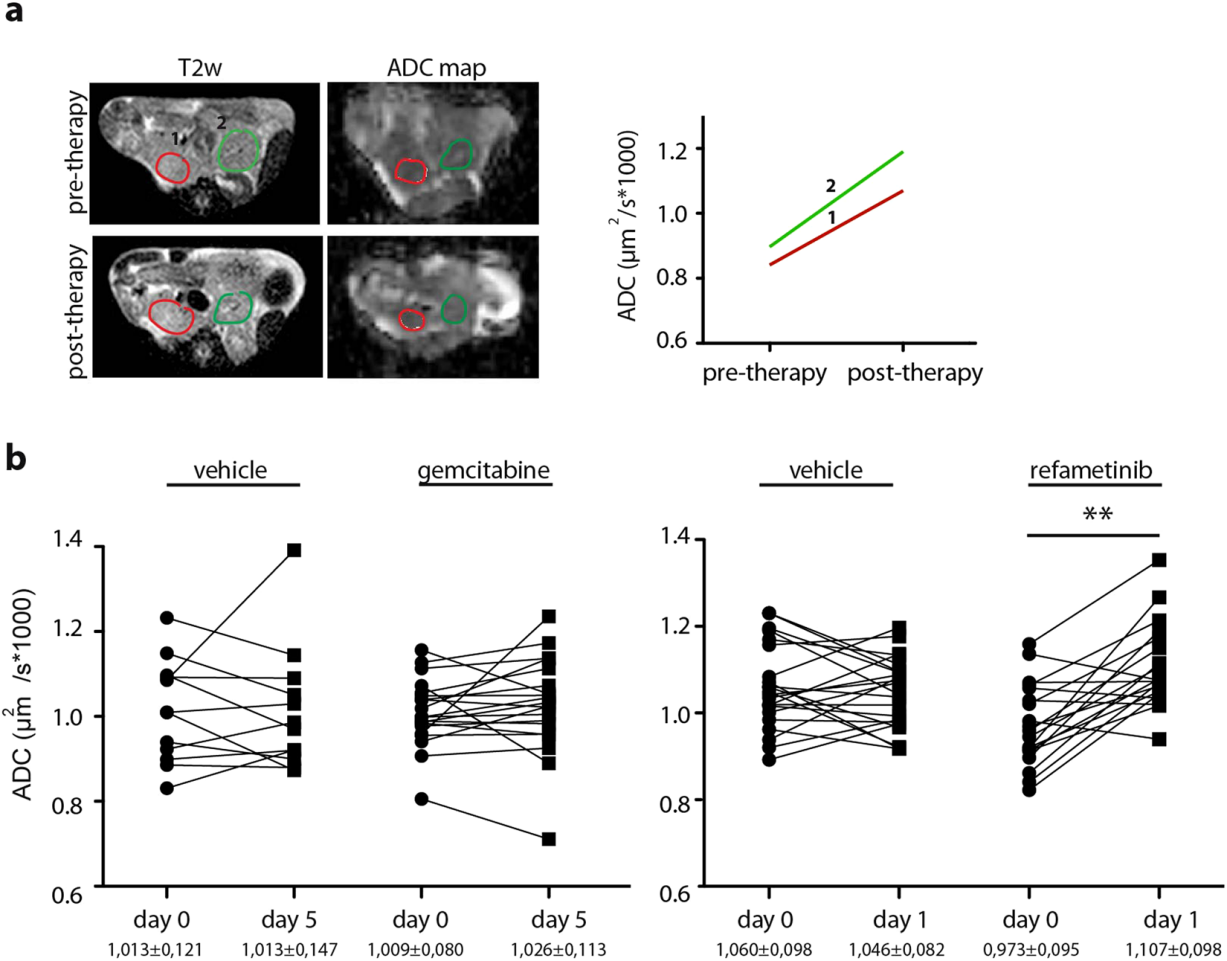
Tumor ADC increases upon refametinib treatment. (**a**) Exemplary presentation of ADC analysis: T2w images together with corresponding ADC maps before and after the therapy (one delineated with red and another delineated with green line). Same tumor was followed pre- and post- therapy and ADC values were calculated. (**b**) Increase of tumor ADC values was observed in refametinib but not in gemcitabine treated animals. One line presents one tumor before and after the therapy. Vehicle p = 0,67; gemcitabine p = 0,21; vehicle p = 0,44; refametinib p = 0,0012.

Similar to [^18^F]-FDG uptake, baseline ADC values in control and treated groups were also variable. Multiple tumor regions in individual CKP mice often presented different ADC values, likely reflecting heterogeneity in PDAC tissue composition (Fig. 3a). However, in a therapy setting, we observed a significant increase in ADC in the refametinib treated group already 24 hours after the first application of the drug. Notably, gemcitabine treatment did not induce changes in ADC value after a short two-course treatment (Fig. 3b) nor after prolonged four-course treatment period (Supplementary Figure 2).

A fixed effect and random effect test analysis was performed for PET and ADC data (Supplementary Table 1).

### Refametinib treatment induces tissue reorganization that is identified by ADC

**Figure 4:**
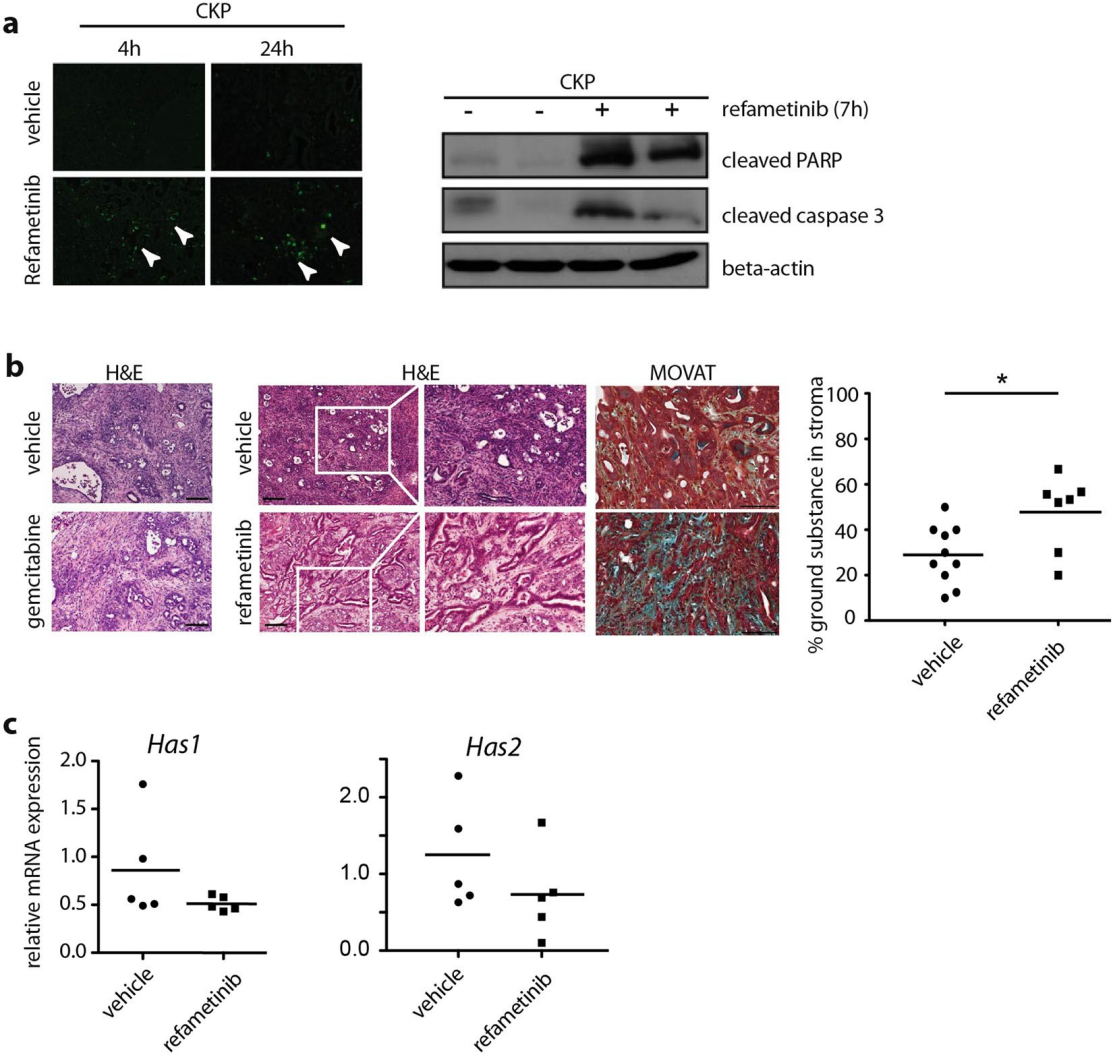
Histological changes induced by refametinib treatment. (**a**) Left panel: TUNEL staining demonstrating apoptosis in refametinib treated mice 4 h and 24 h post-therapy. Right panel: Western blots demonstrating increase in apoptosis markers caspase 3 and cleaved PARP upon refametinib treatment in murine PDAC. (**b**) H&E staining of gemcitabine treated tumor demonstrating no changes in histology while refametinib treated tumors show decrease in cellularity and increase in intercellular space. MOVAT staining and quantification of ground substance demonstrated a ground substance increment in stroma (blue-green color) in refametinib treated tumors. One point presents one tumor. p = 0,027; (**c**). Real-time PCR demonstrates no differences in expression of enzymes involved in synthesis of ground substance, *Has1 and Has2*, among refametinib and vehicle treated tumors. Data normalized to *cyclophilin*.

Refametinib induced massive apoptosis and dramatic histological reorganization of the tumor tissue. TUNEL staining and cleaved PARP/caspase 3 western blot demonstrated induction of apoptosis in the tumors starting only few hours upon refametinib application (Fig. 4a). In the gemcitabine treated group, H&E staining revealed no major histological changes upon treatment. However, refametinib treatment induced a decrease in cellularity and reorganization of stroma that appeared as gain in intercellular space (Fig. 4b). Further stroma analysis with MOVAT pentachrome staining revealed an increase of ground substance content in the stroma (Fig. 4b). Ground substance is a major part of extracellular matrix and mainly composed of glycosoaminoglycans, notably hyaluronic acid. However, semi-quantitative real-time PCR analysis for hyaluronic acid synthases 1 and 2 (*Has1 and Has2*), major enzymes involved in synthesis of hyaluronic acid, demonstrated no significant differences in expression among vehicle and refametinib treated animals (Fig. 4c), suggesting no new synthesis of hyaluronic acid.

### DW-MRI as early therapy response parameter in the clinic

**Figure 5:**
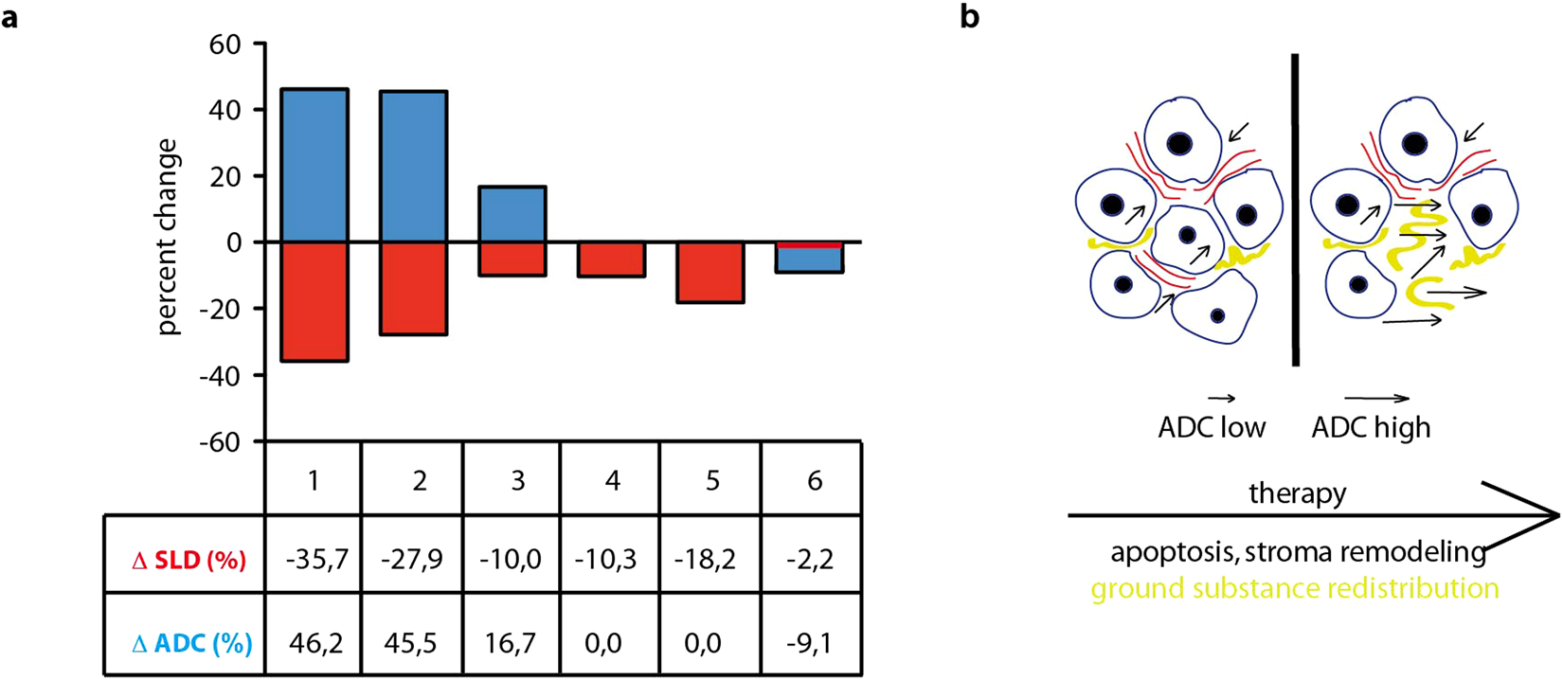
ADC change detects responding patients. (**a**) Patients 1 and 2 show the highest decrease in tumor SLD (sum of the longest diameters) as assessed by CT scan 12 weeks post treatment and the highest increase in ADC value 2 weeks after therapy initiation. Patients were 2 females and 4 males, average age 72,9. (**b**) Proposed model of more intracellular space events through apoptosis and stromal remodeling leading to an increase of ADC values.

Given the promising preclinical data, we set up an exploratory clinical imaging protocol, in which 6 patients with confirmed locally advanced or metastatic, chemotherapy-naïve PDAC were evaluated. Here, DW-MRI before and 2 weeks after initiation of chemotherapy was compared with routinely performed CT-based tumor volume measurements after 8–12 weeks of therapy. Applied chemotherapy protocols were either gemcitabine-based or by the FOLFIRINOX regimen. Notably, the two of the six patients that showed the most pronounced reduction in tumor volume based on CT scans also showed the highest increase in the ADC value (Fig. 5a). While certainly limited by the small size of evaluated patients, these data support further investigation of the ADC parameter as an early response monitoring tool in patients with PDAC.

## Discussion

Advances in therapeutic approaches and molecular subtyping of PDAC^2,3^, impose the necessity for early evaluation of therapy effects in this often rapidly progressing disease. Early therapy-induced effects are probably mirrored in tissue reorganization, changes in cellularity and vascularization or metabolic activity while measurable changes in tumor volume become apparent at later time points if at all. Recent data emphasize the potential of [^18^F]-FDG-PET and DW-MRI in therapy response evaluation. A phase III clinical trial on nab-paclitaxel/gemcitabine in metastatic PDAC suggested that a diminished [^18^F]-FDG uptake could be an early marker of a positive response to therapy^14^. Nishiofuku *et al*.^19^ recently reported that tumor ADC increase during chemotherapy predicts better survival. Similarly, Cuneo *et al*. report that PDACs with lower baseline ADC respond poorly to standard chemotherapy and are candidates for intensified treatment^20^.

To evaluate [^18^F]-FDG-PET and DW-MRI potential for detection of very early therapy response, we performed a preclinical trial in a spontaneous genetic mouse model of PDAC. Therapy was modeled with two different therapeutic regimens, low-active gemcitabine and highly active MEK inhibition with refametinib that was previously shown as active in a xenograft PDAC model^26^ and presented a promising response rate in a recent phase II trial in patients with advanced pancreatic cancer^27^.

Gemcitabine has only a low to absent activity in GEM-based PDAC^22,28^ consistent with our observations (Fig. 1). On the contrary, refametinib led to an almost complete loss of tumor mass only a week after treatment onset. Notably, FDG-PET showed a similar decrease in SUV values to both refametinib and gemcitabine after short treatment (Fig. 2). It is possible that gemcitabine induces transient effects on cancer cell proliferation and thus lower glucose uptake. However, it did not result in a significant decrease in tumor volume as already shown before^28^. Other studies have reported differences in [^18^F]-FDG uptake in tumors treated with two different MEK inhibitors that were both biologicaly active, reflecting the heterogeneity of the metabolic responses^29^. However, and since methodological issues in signal quantification may play an additional role in data interpretation, we cannot at the moment explain why non-active gemcitabine regimen and active MEK inhibition showed similar responses in FDG-PET. To resolve these issues, FDG-PET imaging in therapy response merits further in-depth analysis.

In opposite to FDG-PET, only MEK inhibitor but not gemcitabine-induced effects were observable using DW-MRI showing an increase in ADC values upon refametinib treatment. We recently showed that ADC is a precise and robust determinant of PDAC cellularity and stromal content and that higher ADC values indicate lower cellularity but higher stromal content of the cancer^11^. Furthermore, our work suggested that tumor cellularity is the central restrictor to water diffusivity in the tissue and determinant of the ADC value. ADC values in highly cellular and stroma poor tumors were significantly lower than ADC values in less cellular and stroma rich tumors. Histological analysis showed that refametinib treatment induced a strong apoptotic response and as a consequence a detectable tissue reorganization, more intercellular space and higher presence of ground substance in the stroma (Fig. 4). This potentially relieved the tissue pressure and resulted in less restriction and higher diffusivity, thus reflecting higher ADC values post therapy. As we did not observe an increase in mRNA for enzymes involved in synthesis of ground substance, we assume that in the short period of time of 24 hours post refametinib application no extensive synthesis in the stromal compartment occurs and that increase in ground substance could be attributed to tissue rearrangements.

We evaluated our DW-MRI finding in a small cohort of patients. The preliminary findings here support the principle of an ADC increase in the two best responding patients with the highest tumor volume reduction. Though the data are limited, our observations go in line with similar observations recently published^19,20^ showing an ADC increase as a predictor of increased survival in pancreatic cancer patients treated with chemotherapy.

We are aware that the dramatic treatment response observed upon MEK inhibition in GEM is rarely seen in human PDAC, although the response rate to refametinib treatment in a recent phase II trial was encouraging^27^. However, in our study we did not aim to identify MEK targeting as a successful therapy regimen in terms of overall survival but rather focused on a therapeutic intervention with high initial response rates to validate the ADC parameter as a tool that has the potential to identify individual treatment-responsive patients. We hypothesize that active treatments can lead to decrease in tumor cellularity and rearrangements in the stroma that will reflect to changes in water diffusivity in the tissue and can be detected as changes in ADC value (Fig. 5b). Thus, our results demonstrate that ADC indeed has the potential as a clinically relevant imaging modality for early therapy response evaluation in PDAC and warrants further clinical investigation.

## Methods

### Animal models

*Ptf1a* ^*wt/cre*^; *Kras* ^*wt/LSL-KrasG12D*^; *p53* ^*fl/fl*^ (referred to as CKP) have been previously described^23,24^. All animal protocols were performed according with appropriate guidelines and the experimental protocols were approved by the local Animal Use and Care Committee at the Klinikum Rechts der Isar of the TU München, Germany.

### *In vivo* therapy and monitoring of GEM-based PDAC

For tumor growth kinetics follow-up study, upon T2w-MRI-based tumor detection and enrollment into the study, animals either received gemcitabine (120 mg/kg by intraperitoneal injection, four doses at day 0, 3, 7 and 10) or refametinib (25 mg/kg by oral gavage daily, five days a week). Waterfall graphs presented in Fig. 1 are calculated from tumor volume data obtained after approximately one week of treatment, 5–7 doses of refametinib and two doses of gemcitabine. Volume of the whole tumor was calculated based on T2w images as previously described in detail^23^, Osirix software and in house developed ImageJ-based software that allowed exclusion of cystic non-solid lesions from the calculation. One bar presents one animal.

For imaging studies, upon MRI-based tumor detection and enrollment into the study, animals either received gemcitabine (120 mg/kg by intraperitoneal injection, four doses at day 0, 3, 7 and 10, day 0 considered as therapy start) or refametinib (25 mg/kg by oral gavage, two consecutive days). Vehicles (0, 9% NaCl for gemcitabine trial and 30% HP-CD-Form1 for refametinib trial) were administered accordingly. T2w-MRI, DW-MRI and [^18^F]-FDG-PET were performed for all animals prior to treatment start. Pre-therapy point in imaging studies was performed either at day 0 (directly prior to application of therapy) or one or two days prior to application of therapy, depending on the availability of the MRI and PET scanner. For clarity reasons, pre-therapy point is always labeled as day 0 on the graphs. In gemcitabine or NaCl treated animals, post therapy scans ([^18^F]-FDG-PET and T2w/DW-MRI) were performed on day 4 or 5 (24–48 hours after the second therapy dose), on day 11 ([^18^F]-FDG-PET, 24 hours after the fourth therapy dose) and on day 12 (T2w MRI and DW-MRI, 2 days after the fourth therapy dose). In refametinib and 30% HP-CD-Form1 treated group, post therapy scans (T2w-MRI and DW-MRI) were performed 24 hours after single administration of the drug; a second dose was then administered and [^18^F]-FDG-PET performed 24 hours after the second dose.

### Histology, Immunohistochemistry, Western Blot

Animals were sacrificed upon reaching defined endpoint criteria in survival studies or after a planned imaging protocol was finished. Tissues were either cryo-fixed or whole mount fixed in 4% PFA 24–48 h, transferred to PBS and paraffin embedded. H&E was performed on tissue samples according to standard protocols. MOVAT pentachrome staining (modified according to Verhoeff) was performed manually using a staining kit (Morphisto GmbH, Frankfurt, Germany). Histological evaluation was performed by two expert pathologists (K.S. and I.E.). H&E and MOVAT was performed on tissues from animals treated with refametinib or vehicle for two consecutive days and tissues were collected 24 hours post last application. H&E was performed on tissues from gemcitabine and vehicle treated animals that were sacrificed 1–4 days post after last (4^th^) gemcitabine/vehicle injection. For Western blot, refametinib treated tumor tissue was collected 7 hours post treatment. Western blot was performed according to standard protocols with antibodies against cleaved PARP and cleaved caspase 3 (Cell Signaling). Real-time PCR was performed according to the standard protocols on Roche Light Cycler 480. *Cyclophilin A* and *XS13* were used as housekeeping genes. Primer sequences for *Has1* and *Has2* are taken from Calve *et al*.^30^.

### Imaging protocols and data analysis for preclinical study

Detailed description of imaging procedure can be found in Heid *et al*.^11^. For MRI, tumor growth kinetics changes were followed with T2 weighted imaging protocol using a 47-mm *microscopy* surface *coil* inside a Philips Achieva 1, 5 T clinical scanner (Achieva 1.5 T, Philips Medical Systems, Best, The Netherlands). An axial multi-slice T_2_-weighted (T2w) TSE sequence (resolution 0.3 × 0.3 × 0.7 mm^3^, minimum 30 slices, TE = 90 ms, TR > 3 s) was applied for tumor detection. Solid tumor volumes were calculated using in house optimized ImageJ based software that differentiates between solid and cystic parts of the tumor. Following the anatomy scan, an axial multi-slice DW-MRI sequence covering the tumors was performed (resolution 0.7 × 0.7 × 1.5 mm^3^, EPI factor = 45, TR/TE = 3000/60 ms, b_0-2_ values = 20, 200, 600 s/mm^2^, averages = 10). An ADC map was calculated using in house adapted IDL software with the equation: ADC = ln(S_2_/S_i_)/(b_i_ − b_2_), with (i = 0, 1) and S_2_ (b_2_ = 600 s/mm^2^), S_1_ (b_1_ = 200 s/mm^2^), S_0_ (b_0_ = 20 s/mm^2^).

For PET, tumor-bearing CKP animals were i.v. injected with 7–12 µBq [^18^F]-FDG after fasting for at least 6 hours and scanned on a small-animal PET/CT scanner (Inveon, SIEMENS Preclinical Solutions, Knoxville, TN). Images were analyzed using the Inveon Research Workplace (Siemens Preclinical Solutions, Knoxville, TN, USA). 3-dimensional disc Region of Interest (ROI)s at the thickness of MRI measurements (1.5 mm) corresponding to ADC-map drawn ROI were depicted on the PET images. Mean tumor standard uptake value of all voxel in the ROI (SUVmean) was calculated as a ratio of tissue radioactivity concentration found in the ROI and administered dose corrected by mouse weight^31^.

ROIs were manually defined based on histology and quality of imaging data. A minimum of five animals was followed in each group. For all murine imaging analysis two to three different tumor regions (when scan quality allowed, one in the head, one in the neck and one in tail of the pancreas) were analyzed pre and post therapy per mouse. Attention was payed that ROI’s are positioned on same/similar anatomical position on pre- and post-therapy scans as much as that was technically allowed (changes due to animal positioning, bowel movements, breathing). After fusion of MRI and PET imaging data sets (IRW, Siemens, Erlangen), H&E slices were manually co-registered with the corresponding T2w anatomical imaging data. Tumor regions were identified in at least 3 axial H&E stained slices per tumor, each tumor region covering a thickness of approximately 1.2 mm dehydrated tissue. ROIs (1.5 mm slice thickness) were defined in T2w images and transferred to ADC maps and PET data.

### Patient Treatment and imaging

Patients with chemotherapy-naïve stage IV PDAC and measurable disease documented by computed tomography (CT) imaging received either gemcitabine-based chemotherapy or the FOLFIRINOX regimen^32^ at the physician’s discretion. Tumor volume assessment by CT scans (RECIST version 1.1, sum of the longest diameters (SLD) representing tumor load) were performed 8–12 weeks after therapy initiation as per clinical routine.

### MRI in humans

The study protocol was approved by the ethics committee of the Faculty of Medicine at TU München, Germany (document no. 374/15). Written informed consent was obtained from all patients. All methods were performed in accordance with the relevant guidelines and regulations. Detailed description of imaging procedure can be found in Heid *el al*.^11^.

### Statistical Analysis

For PET SUV and ADC analysis, a Wilcoxon matched pairs test was used. Mann-Whitney test was used for MOVAT and real-time PCR analysis. Statistical analysis was conducted using GraphPad Prism version 5. A fixed effect and random effect test was performed in R (Lme4 package).

### Data Availability

The datasets generated during and/or analyzed during the current study are available from the corresponding author upon request.

## Electronic supplementary material

Supplementary Figures

Supplementary Tables

## References

[1] Ryan DP, Hong TS, Bardeesy N (2014). Pancreatic adenocarcinoma. N Engl J Med.

[2] Gostimir M, Bennett S, Moyana T, Sekhon H, Martel G (2016). Complete pathological response following neoadjuvant FOLFIRINOX in borderline resectable pancreatic cancer - a case report and review. BMC Cancer.

[3] Hartlapp I (2013). Complete pathological remission of locally advanced, unresectable pancreatic cancer (LAPC) after intensified neoadjuvant chemotherapy. Onkologie.

[4] Collisson EA (2011). Subtypes of pancreatic ductal adenocarcinoma and their differing responses to therapy. Nat Med.

[5] Bailey P (2016). Genomic analyses identify molecular subtypes of pancreatic cancer. Nature.

[6] Epelbaum R (2013). Tumor aggressiveness and patient outcome in cancer of the pancreas assessed by dynamic 18F-FDG PET/CT. J Nucl Med.

[7] Rosenkrantz AB, Matza BW, Sabach A, Hajdu CH, Hindman N (2013). Pancreatic cancer: lack of association between apparent diffusion coefficient values and adverse pathological features. Clin Radiol.

[8] D’Onofrio M (2013). Perfusion CT can predict tumoral grading of pancreatic adenocarcinoma. Eur J Radiol.

[9] Park MJ (2014). Preoperative detection of small pancreatic carcinoma: value of adding diffusion-weighted imaging to conventional MR imaging for improving confidence level. Radiology.

[10] Lee ES, Lee JM (2014). Imaging diagnosis of pancreatic cancer: a state-of-the-art review. World J Gastroenterol.

[11] Heid I (2017). Co-clinical Assessment of Tumor Cellularity in Pancreatic Cancer. Clin Cancer Res.

[12] Kim JY (2013). Utilisation of combined 18F-FDG PET/CT scan for differential diagnosis between benign and malignant adrenal enlargement. Br J Radiol.

[13] Asagi A (2013). Utility of contrast-enhanced FDG-PET/CT in the clinical management of pancreatic cancer: impact on diagnosis, staging, evaluation of treatment response, and detection of recurrence. Pancreas.

[14] Ramanathan RK (2016). Positron emission tomography response evaluation from a randomized phase III trial of weekly nab-paclitaxel plus gemcitabine versus gemcitabine alone for patients with metastatic adenocarcinoma of the pancreas. Ann Oncol.

[15] Koh DM, Collins DJ (2007). Diffusion-weighted MRI in the body: applications and challenges in oncology. AJR Am J Roentgenol.

[16] De Robertis R (2015). Diffusion-weighted imaging of pancreatic cancer. World J Radiol.

[17] Ma X (2014). Quantified ADC histogram analysis: a new method for differentiating mass-forming focal pancreatitis from pancreatic cancer. Acta Radiol.

[18] Del Chiaro M (2015). Short-term Results of a Magnetic Resonance Imaging-Based Swedish Screening Program for Individuals at Risk for Pancreatic Cancer. JAMA Surg.

[19] Nishiofuku H (2016). Increased tumour ADC value during chemotherapy predicts improved survival in unresectable pancreatic cancer. Eur Radiol.

[20] Cuneo KC (2014). A pilot study of diffusion-weighted MRI in patients undergoing neoadjuvant chemoradiation for pancreatic cancer. Transl Oncol.

[21] Singh M (2010). Assessing therapeutic responses in Kras mutant cancers using genetically engineered mouse models. Nat Biotechnol.

[22] Ardito CM (2012). EGF receptor is required for KRAS-induced pancreatic tumorigenesis. Cancer Cell.

[23] Mazur PK (2015). Combined inhibition of BET family proteins and histone deacetylases as a potential epigenetics-based therapy for pancreatic ductal adenocarcinoma. Nat Med.

[24] Bardeesy N (2006). Bothp16(Ink4a) and the p19(Arf)-p53 pathway constrain progression of pancreatic adenocarcinoma in the mouse. Proc Natl Acad Sci USA.

[25] Mazur PK, Siveke JT (2012). Genetically engineered mouse models of pancreatic cancer: unravelling tumour biology and progressing translational oncology. Gut.

[26] Chang Q, Chapman MS, Miner JN, Hedley DW (2010). Antitumour activity of a potent MEK inhibitor RDEA119/BAY 869766 combined with rapamycin in human orthotopic primary pancreatic cancer xenografts. BMC Cancer.

[27] Van Laethem JL (2017). Phase I/II Study of Refametinib (BAY 86-9766) in Combination with Gemcitabine in Advanced Pancreatic cancer. Target Oncol.

[28] Olive KP (2009). Inhibition of Hedgehog signaling enhances delivery of chemotherapy in a mouse model of pancreatic cancer. Science.

[29] Kraeber-Bodere F (2012). Differences in the biologic activity of 2 novel MEK inhibitors revealed by 18F-FDG PET: analysis of imaging data from 2 phase I trials. J Nucl Med.

[30] Calve S, Isaac J, Gumucio JP, Mendias CL (2012). Hyaluronic acid, HAS1, and HAS2 are significantly upregulated during muscle hypertrophy. Am J Physiol Cell Physiol.

[31] Thie JA (2004). Understanding the standardized uptake value, its methods, and implications for usage. J Nucl Med.

[32] Conroy T (2011). FOLFIRINOX versus gemcitabine for metastatic pancreatic cancer. N Engl J Med.

