# Supplementary material for: Apparent Diffusion Coefficient (ADC) predicts therapy response in pancreatic ductal adenocarcinoma
Source: Sci Rep. 2017 Dec 6;7:17038. doi: 10.1038/s41598-017-16826-z (PMC5719052; doi:10.1038/s41598-017-16826-z)
Supplement: Supplementary file 1 — Supplementary Figures [file 41598_2017_16826_MOESM1_ESM.doc]

**Apparent Diffusion Coefficient (ADC) predicts therapy response in pancreatic ductal adenocarcinoma**

**Short title:** Diffusion-weighted magnetic resonance imaging in pancreatic cancer.

M. Trajkovic-Arsic^1,2,3^‡, I. Heid^4^‡, K. Steiger^5^, A. Gupta^3^, A. Fingerle^4^, C. Wörner^4^, N. Teichmann^3^, S. Sengkwawoh-Lueong^1,2^, A.J. Beer^6#^, I. Esposito^5^*, R. Braren^4^* and J.T.Siveke^1,2,3^*

‡ equally contributed, * corresponding authors

**Supplementary figure 1:**

**
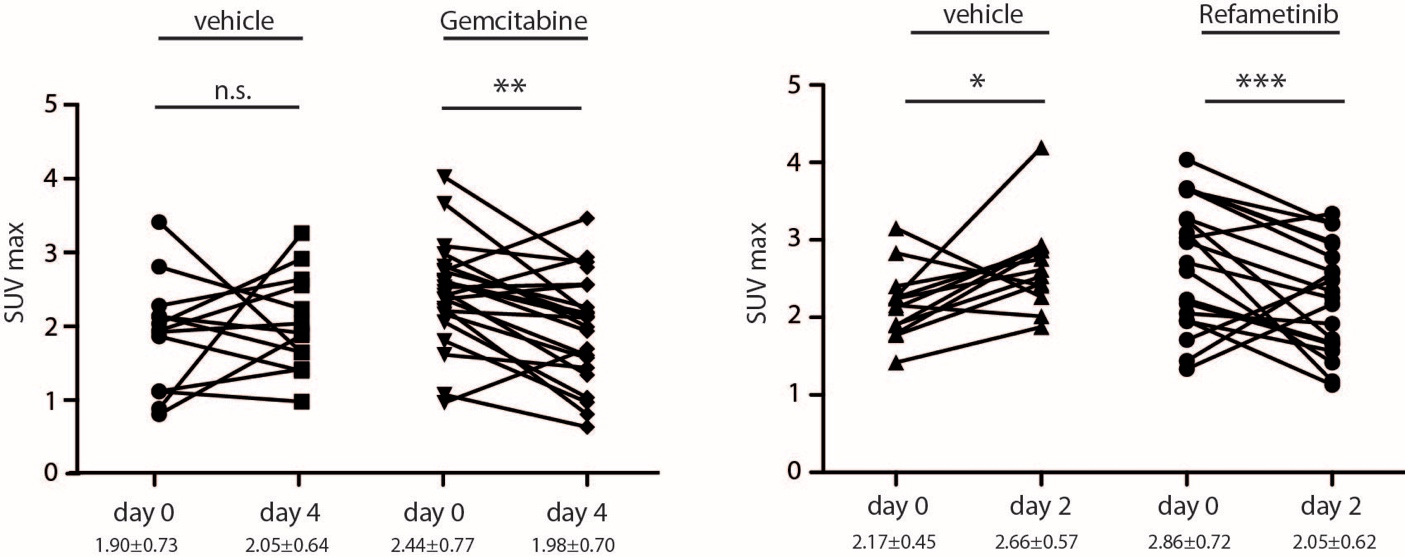
**

SUVmax analysis of gemcitabine/control and refametinib/control animals pre- and post-therapy. Vehicle p=0.6355; gemcitabine p=0.0045; vehicle 0.032; refametinib p=0.0009.

**Supplementary figure 2:**

**
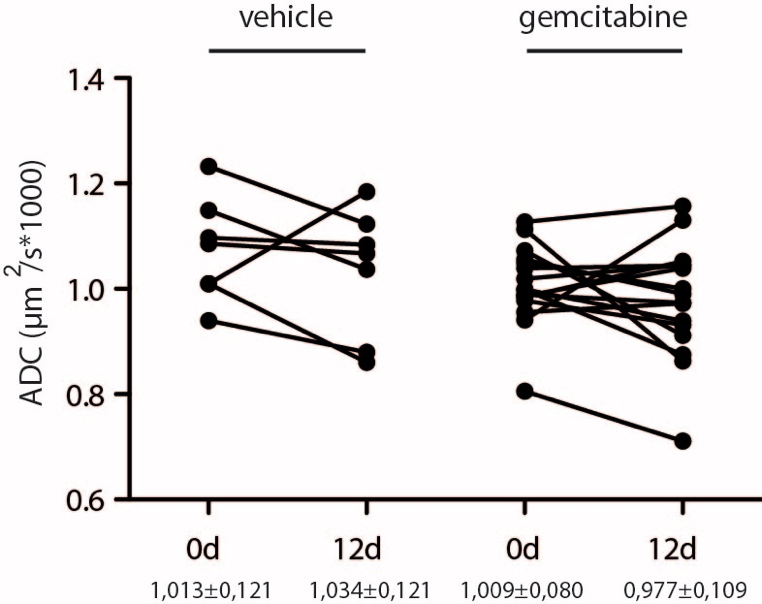
**

No change in ADC values upon prolonged 4-dose treatment with gemcitabine in CKP tumors. One line presents one tumor before and after therapy. Vehicle p=0.269; gemcitabine p=0.289.
