# Supplementary material for: Apparent Diffusion Coefficient (ADC) predicts therapy response in pancreatic ductal adenocarcinoma
Source: Sci Rep. 2017 Dec 6;7:17038. doi: 10.1038/s41598-017-16826-z (PMC5719052; doi:10.1038/s41598-017-16826-z)
Supplement: Supplementary file 2 — Supplementary Tables [file 41598_2017_16826_MOESM2_ESM.docx]

|  | Variable | Coif | F-test | P |
| --- | --- | --- | --- | --- |
| SUVmean_Ref/veh | Intercept | 1.69±0.13 | 797.08 | <0.0001 |
|  | Time | 0.37±0.17 | 8.58 | 0.006 |
|  | Treatment | 0.45±0.17 | 0.004 | 0.97 |
|  | Time:Treatment | -0.97±0.22 | 33.40 | <0.0001 |
|  |  |  |  |  |
| SUVmean_Gem/veh | Intercept | 1.49±0.15 | 462.7 | <0.0001 |
|  | Time | 0.12±0.15 | 6.1 | 0.018 |
|  | Treatment | 0.46±0.18 | 1.3 | 0.26 |
|  | Time:Treatment | -0.55±0.18 | 8.5 | 0.006 |
| ADC_Gem/veh |  |  |  |  |
|  | Intercept | 1.01±0.03 | 3285 | <0.0001 |
|  | Time | 0.0003±0.03 | 0.34 | 0.56 |
|  | Treatment | -0.004±0.04 | 0.013 | 0.91 |
|  | Time:Treatment | 0.02±0.04 | 0.21 | 0.65 |
| ADC_Ref/veh |  |  |  |  |
|  | Intercept | 1.06±0.02 | 7486 | <0.0001 |
|  | Time | -0.01±0.02 | 11.3 | 0.002 |
|  | Treatment | -0.08±0.03 | 0.3 | 0.6 |
|  | Time:Treatment | 0.15±0.03 | 20.6 | 0.0001 |
| SUVmax_Ref/veh |  |  |  |  |
|  | Intercept | 2.17±0.17 | 694.9 | <0.0001 |
|  | Time | 0.49±0.19 | 4.84 | 0.035 |
|  | Treatment | 0.69±0.22 | 0.04 | 0.84 |
|  | Time:Treatment | -1.31±0.25 | 28.73 | <0.0001 |
| SUVmax_Gem/veh |  |  |  |  |
|  | Intercept | 1.89±0.19 | 488.8 | <0.0001 |
|  | Time | 0.15±0.20 | 3.6 | 0.07 |
|  | Treatment | 0.54±0.24 | 1.4 | 0.25 |
|  | Time:Treatment | -0.61±0.26 | 5.5 | 0.02 |

Explanatory effects of fixed variables

|  | Tumour | Model | Loglik | Test | LRT | df | P |
| --- | --- | --- | --- | --- | --- | --- | --- |
| SUVmean_Ref/veh |  | 1 | -40.37 |  |  |  |  |
|  | x | 2 | -42.05 | 1 vs 2 | 1.4 | 6 | 0.24 |
| SUVmean_Gem/veh |  | 1 | -58.17 |  |  |  |  |
|  | x | 2 | -62.91 | 1 vs 2 | 9.5 | 6 | 0.0021 |
| ADC_Gem/veh |  | 1 | 46.6 |  |  |  |  |
|  | x | 2 | 40.4 | 1 vs 2 | 12.4 | 6 | 4.00E-04 |
| ADC_Ref/veh |  | 1 | -116.2 |  |  |  |  |
|  | x | 2 | -114.6 | 1 vs 2 | 5.9 | 6 | 0.01 |
| SUVmax_Ref/veh |  | 1 | -57.08 |  |  |  |  |
|  | x | 2 | -59.65 | 1 vs 2 | 5.12 | 6 | 0.02 |
| SUVmax_Gem/veh |  | 1 | -73.84 |  |  |  |  |
|  | x | 2 | -76.86 | 1 vs 2 | 6.02 | 6 | 0.01 |

Explanatory effects of random effects
